# Prolonged antibiotic prophylaxis use in elective orthopaedic surgery – a cross-sectional analysis

**DOI:** 10.1186/s12891-021-04290-w

**Published:** 2021-05-06

**Authors:** Felix Rohrer, Anita Maurer, Hubert Noetzli, Brigitta Gahl, Andreas Limacher, Tanja Hermann, Jan Bruegger

**Affiliations:** 1 Department of Internal Medicine, Sonnenhofspital, 3006 Bern, Switzerland; 2 Centre Hospitalier Universitaire Vaudois, CHUV, 1011 Lausanne, Switzerland; 3 University of Bern, 3012 Bern, Switzerland; 4 Orthopaedie Sonnenhof, 3006 Bern, Switzerland; 5 Clinical Trials Unit (CTU) Bern, University of Bern, 3012 Bern, Switzerland; 6 Stiftung Lindenhof, Campus SLB, Swiss Institute for Translational and Entrepreneurial Medicine, 3010 Bern, Switzerland; 7 University of Zurich, 8006 Zurich, Switzerland

**Keywords:** Surgical antibiotic prophylaxis, Prolonged surgical antibiotic prophylaxis, Surgical site infection, Orthopaedic surgery, Elective surgery, Prevention

## Abstract

**Purpose:**

Surgical antibiotic prophylaxis (SAP) prevents surgical site infections (SSI). In orthopaedic surgery, the use of prolonged SAP (PSAP) has been reported in daily routine, despite guidelines advising against it. Therefore, we asked: What is the proportion of PSAP use, defined as administration of SAP ≥24 h after elective orthopaedic surgery? Are there patient- and surgery-related predictors of PSAP use?

**Methods:**

This cross-sectional analysis investigated 1292 patients who underwent elective orthopaedic surgery including total joint arthroplasties at one Swiss centre between 2015 and 2017. Patient comorbidities, surgical characteristics and occurrence of SSI at 90 days in PSAP group were compared to the SAP group (< 24 h post-operative).

**Results:**

PSAP use was 12% (155 of 1292). Patient-related factors associated with PSAP compared to the SAP group included older age (63 vs. 58y; *p* < 0.001), higher BMI (29 vs. 27 kg/m^2^; *p* < 0.001), ASA classification ≥3 (31% vs. 17%; *p* < 0.001) and lung disease (17% vs. 9%; *p* = 0.002). Surgery-related factors associated with PSAP were use of prosthetics (62% vs. 45%; *p* < 0.001), surgery of the knee (65% vs. 25%; *p* < 0.001), longer surgery duration (87 vs. 68 min; *p* < 0.001) and presence of drains (90% vs. 65%; *p* < 0.001). All four SSI occurred in the SAP group (0 vs. 4; *p* = 1.0). Surgeons administered PSAP with varying frequencies; proportions ranged from 0 to 33%.

**Conclusion:**

PSAP use and SSI proportions were lower than reported in the literature. Several patient- and surgery-related factors associated with PSAP use were identified and some were potentially modifiable. Also, experienced surgeons seemed to implement differing approaches regarding the duration of SAP administration.

## Introduction

Surgical site infections (SSI) can have devastating consequences for patients. They are associated with inferior patient outcomes as well as a high economic burden [1–4]. SSI proportions are reported to be 1–5% in patients undergoing major surgery [4–6]. Up to 55% of SSI are estimated to be preventable with the use of evidence- based strategies, including appropriate use of surgical antibiotic prophylaxis (SAP) [7]. Although SAP is an effective prevention measure [8], a safe use of SAP is needed to prevent potential resistances and adverse effects of antibiotics [9–11]. Heterogenous guidelines reflect the ongoing discussion about the optimal duration of SAP. Several recommend discontinuing SAP within 24 h after operation [12–14]. Besides that, the 2017 U.S. Centers for Disease Control and Prevention (CDC) guideline even recommend against administration of SAP after closure of the operation site in all clean or clean-contaminated procedures [15]. Nevertheless, recommendations on shortened SAP remain a matter of controversy, especially in conditions with potentially higher risk for SSI - including presence of a wound drain [16] - or prosthetic procedures with high risk for devastating outcomes if SSI occurs [17, 18]. A retrospective cohort study found patients with total hip or knee arthroplasties (THA/TKA) to be 4–5 times more likely to develop a periprosthetic joint infection (PJI) if they were not administered extended oral SAP [19]. On the other hand, a recently published meta- analysis of 51,627 total joint arthroplasties (TJA) found no added benefit of prolonged antibiotic prophylaxis (PSAP, defined as administration ≥24 h post-operative) [20]. Therefore, the shortest effective and safe duration of SAP in SSI prevention in orthopaedic surgery remains a topic of debate.

Implementation and adherence to guidelines is reportedly insufficient and non-adherence may even lead to higher SSI proportions [6, 21, 22]. Furthermore, most studies in the literature evaluate SSI and SAP in patients undergoing either prosthetic surgery of the hip or knee [8, 16, 18, 21, 23–26], knee arthroscopy [27, 28] or spinal surgery [29–31]. To our knowledge, no study has reported SAP use and SSI proportions in a broad population undergoing elective orthopaedic surgery.

## Methods

### Aim

What is the proportion of PSAP use, defined as administration of SAP ≥24 h after elective orthopaedic surgery? Are there patient- and surgery-related predictors for PSAP use?

### Study design

This study is a cross-sectional analysis of elective orthopaedic operations between November 2015 and September 2017 at one tertiary care centre in Bern, Switzerland. The study protocol was approved by the local ethics committee (PB_2016_00256). Written informed consent was obtained from each patient.

### Data collection

Patient characteristics and occurrence of SSI were prospectively surveyed. Other data (surgical characteristics, antibiotic prophylaxis) was retrospectively extracted from the electronic patient file system (KISIM, Cistec AG, Zurich, Switzerland). All relevant data was entered into the secure web data storing system REDCap (Research Electronic Data Capture, Version 8.5.19, Vanderbilt University, Nashville, Tennessee, USA).

### Participants

Patients for this study were initially recruited for the randomized controlled trial (RCT) DECO- SSI (DECOlonisation and SSI), which investigated the impact of pre-operative decolonization on the occurrence of SSI [32]. Inclusion criteria were a minimum age of 16 years and a timeframe of at least 14 days before surgery. Each patient was included in the study only once and for only one operation. Exclusion criteria were: allergy to mupirocin or chlorhexidine, the presence of a foreign nasal body, pregnancy or planned intervention for a documented infection. During the DECO-SSI study, all patients underwent pre-operative screening for *Staphylococcus aureus* by nose swab.

**Fig. 1:**
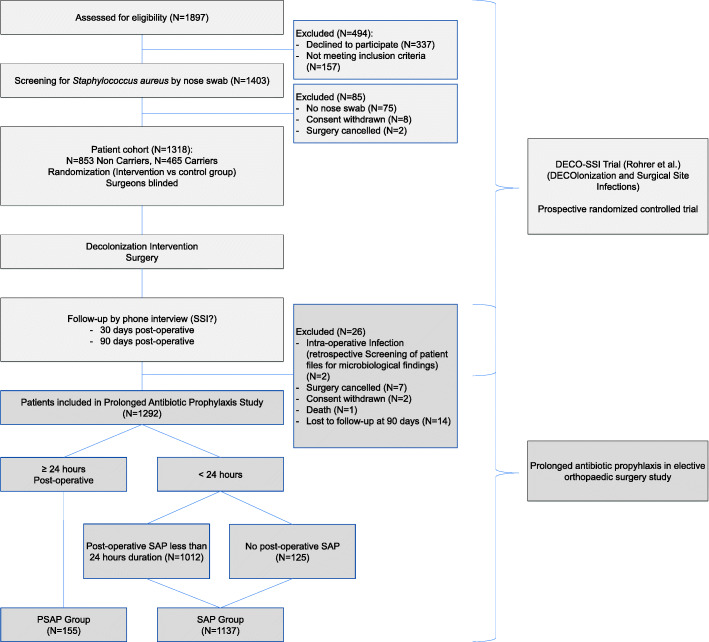
Patient flow chart

In this study, patients were grouped according to the duration of administered SAP, irrespective of their *S. aureus* carrier status. The SAP group received antibiotic prophylaxis for a duration shorter than 24 h; including those receiving no post-operative SAP at all and those with SAP discontinued within 24 h after the operation. The PSAP group consisted of patients who received SAP for 24 h or longer (Fig. 1). The standard SAP at our institution is Cefuroxime intravenous, one dose given 0-60 min before incision with or without two doses administered 8 h and 16 h post-operative. Clindamycin is used in case of allergy. Prescription of differing SAP or duration remained the responsibility of the treating surgeon, depending on clinical assessment. Possible deviations are analysed in this study.

### Outcomes

Primary outcome was the number of patients receiving PSAP, defined as administration of SAP ≥24 h post-operative. The number of additional doses, dosing regimen, route of administration and duration of post-operative SAP were collected. To ensure that PSAP was not prescribed for therapeutic reasons, all cases with PSAP were reviewed by an internal medicine physician. Patient files were screened for surgery site microbiological samples taken peri-operatively and for reasons of antibiotic use other than prophylaxis; namely for treatment of an infection at surgical site, hospital- acquired or independent of surgery and hospital setting. Two patients with findings consistent with an intra-operative infection were excluded from this analysis.

Potential predictors for PSAP were the following: patient comorbidities and surgical characteristics including type of anaesthesia, use of foreign material, operating surgeon, drain use and post-operative transfusions. Secondary outcome was occurrence of SSI, which was prospectively surveyed as part of the DECO-SSI trial by telephone interview at 30 and 90 days [32]. SSI was defined by CDC criteria [33].

### Statistical analysis

Statistical analysis was performed by an independent statistician from the clinical trials unit, University of Bern, Switzerland. Continuous variables are shown as mean with standard deviation. Comparisons were made using Students T-test. Categorical data is shown as number (%) and compared using Fisher’s exact test. We calculated odds ratios (OR) with 95% confidence intervals to quantify the association of patient characteristics and surgery details with outcome using logistic regression. In case of zero cells, we applied a continuity correction of 0.5. We used logistic regression to investigate whether experienced surgeons administered PSAP with the same frequencies included the use of foreign material as covariate. Only surgeons who had carried out > 50 operations on patients in the study cohort were included in this analysis. Based on surgeons’ proportion of operations followed by PSAP, we selected a surgeon who had neither the highest nor the lowest proportion as a reference to derive OR. We compared the model fit of the logistic regression with only surgeons with the model fit when use of foreign material was included as covariate using the likelihood ratio test. All other analyses related to the entire study cohort. All analyses were carried out using Stata 16 (Stata Corp., College Station, Texas).

## Results

A total of 1292 patients were included in this analysis (Fig. 1). Patient average age was 58 years (±14) with slightly more females (53%, 682 of 1292). Hip, knee and spinal surgery accounted for most operations (424 (33%), 384 (30%) and 239 (18%) of 1292, respectively). Prosthetic surgery accounted for 47% (613 of 1292).

**Fig. 2:**
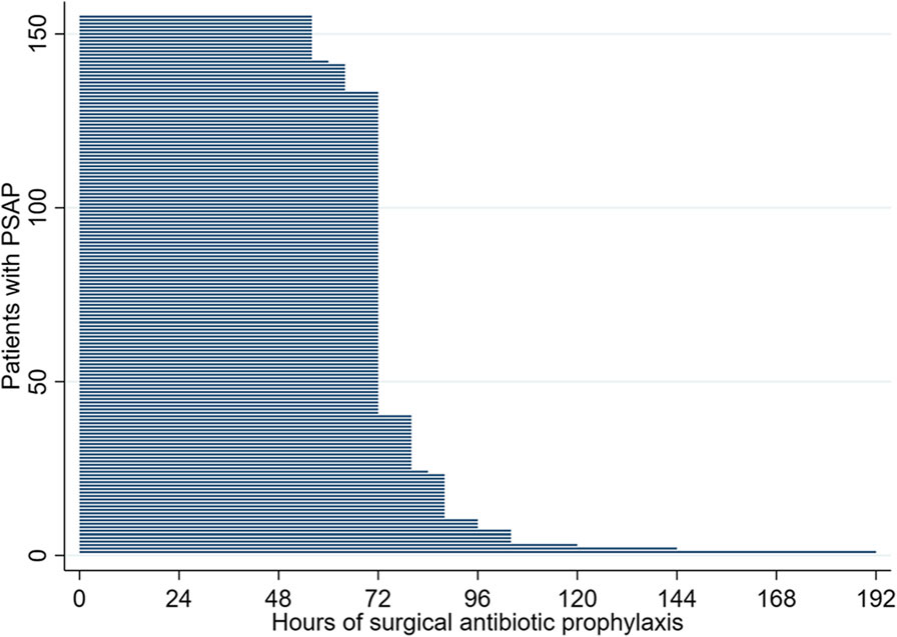
Duration of post-operative surgical antibiotic prophylaxis in PSAP group (*N* = 155) is shown in this plot

PSAP use was 12% (155 of 1292). Duration of PSAP was additional 3.3 (±1.5) doses ≥24 h after operation. This results in additional 27 (±15) hours of antibiotic prophylaxis (Table 1, Fig. 2). In the SAP group, 11% (125 of 1137 patients) did not receive any post-operative SAP.

**Table 1:** Antibiotic prophylaxis

Characteristics	SAP (***N*** = 1137)	PSAP (***N*** = 155)	***P***-Value
Antibiotic agent			0.003
Cefuroxime	1000 (88%)	147 (95%)	
Vancomycin	4 (0.35%)	3 (1.9%)	
Clavulanic acid/ amoxicillin	0 (0.00%)	1 (0.65%)	
Clindamycin	8 (0.70%)	4 (2.6%)	
Number of consecutive post-operative intake doses < 24 h (number of patients)			
0	125 (11%)	0	
1	12 (1.1%)	0	
2	999 (87.8%)	155 (100%)^a^	
3	1 (0.1%)	0	
Route of administration < 24 h			1.00
IV (intravenous)	1006 (88%)	154 (99%)	
p.o. (peroral)	6 (0.53%)	1 (0.65%)	
Number of consecutive intake doses ≥24 h (number of doses)	n/a	3.3 (1.5)	
Route of administration ≥24 h			
IV (intravenous)	n/a	152 (98%)	
p.o. (peroral)	n/a	3 (1.9%)	
Antibiotic prophylaxis duration ≥24 h after surgery (in hours)^b^	n/a	27 (15)	
Presence of wound drain installed during operation	740 (65%)	139 (90%)	< 0.001
Number of drains in surgical site	1.1 (0.27)	1.5 (0.53)	< 0.001
Number of drains in surgical site			< 0.001
1	686 (60%)	74 (48%)	
2	53 (4.7%)	63 (41%)	
3	1 (0.09%)	2 (1.3%)	
Drain 1 - Duration of drain (in hours)	26 (11)	43 (14)	< 0.001
Drain 2 - Duration of drain (in hours)	32 (16)	44 (10)	< 0.001
Cumulative drain output (of all drains) (in ml)	131 (147)	224 (223)	< 0.001
Post-operative transfusion of concentrated red blood cells	11 (0.97%)	2 (1.3%)	0.66
Other transfusion (platelet concentrate, fresh frozen plasma, albumin)	3 (0.26%)	0 (0.00%)	1.00
Record of NRS (Nutritional-Risk-score)^c^	247 (22%)	50 (32%)	0.004
Absolute NRS score	1.9 (0.85)	2.1 (1.0)	0.13
Surgical site infection	4 (0.35%)	0 (0.00%)	1.00

Footnote: Numbers are shown as mean (sd) or n (%)

^a^All patients receiving PSAP, i. e. surgical antibiotic prophylaxis for more than 24 h, also received 2 doses of SAP in the first 24 h post-operative

^b^Antibiotic prophylaxis duration ≥24 h after surgery (in hours) = number of consecutive intake doses multiplied with time interval between doses in hours

^c^NRS score: screening tool for malnutrition. Internal nursing guidelines recommend collection of NRS score: if a stay ≥72 h is expected or one of the following criteria is met: Patient ≥70 years old (NRS score at third day of hospitalization), BMI < 20 kg/m2 (NRS score at admission), patients with loss of appetite or apparent malnutrition (NRS score at admission), patients undergoing major visceral operation (NRS score at third day of hospitalization), patients with known tumor disease (NRS score at third day of hospitalization), patients undergoing chemotherapy (NRS score at admission), all patients at eighth day of hospitalization. If NRS score is < 3 points weekly reevaluation is proceeded. NRS score ≥ 3 points needs intervention. NRS score ≥ 3 points is considered as light, = 4 points moderate and ≥ 5 points substantial energy and protein malnutrition

PSAP use was associated with older age (63 vs. 58 years; *p* < 0.001; OR 1.03/year), higher body mass index (BMI) (29 vs. 27 kg/m2; *p* < 0.001; OR 1.06), American Society of Anesthesiologists (ASA) classification ≥3 (31% vs. 17%; *p* < 0.001; OR 1.6), known congestive or ischemic heart disease (10% vs 6%; *p* = 0.05; OR 1.84) and known lung disease (17% vs. 9%; *p* = 0.002; OR 2.06) (Table 2). Screening for malnutrition with the nutritional-risk-score (NRS) was available for 23% of patients with a mean absolute value of 1.9 (±0.88). Indications for screening are listed in Table 1. NRS was significantly more frequently available for patients with PSAP, whereby the score did not differ between PSAP and SAP (2.1 ± 1.0 vs. 1.9 ± 0.85; *p* = 0.31).

**Table 2:** Patient characteristics

Characteristics	SAP (***N*** = 1137)	PSAP (***N*** = 155)	***P***-value	OR (95% CI)
Age (years)	58 (14)	63 (12)	< 0.001	1.03 (1.02 to 1.04)
Sex (female)	603 (53%)	79 (51%)	0.67	0.92 (0.66 to 1.29)
Active Smoking	206 (18%)	17 (11%)	0.031	0.56 (0.33 to 0.94)
Alcohol intake > 2 units/ day^a^	32 (2.8%)	3 (1.9%)	0.45	0.68 (0.21 to 2.25)
BMI (kg/m^2)	27 (4.8)	29 (5.6)	< 0.001	1.06 (1.03 to 1.10)
^b^Lung Disease	101 (8.9%)	27 (17%)	0.002	2.16 (1.36 to 3.44)
COPD	17 (1.5%)	5 (3.2%)	0.17	2.20 (0.80 to 6.04)
Asthma	36 (3.2%)	13 (8.4%)	0.005	2.80 (1.45 to 5.41)
Other severe lung disease	56 (4.9%)	17 (11%)	0.005	2.38 (1.34 to 4.21)
Congestive or ischemic heart disease	67 (5.9%)	16 (10%)	0.05	1.84 (1.04 to 3.26)
Liver disease	10 (0.88%)	0 (0.00%)	0.62	0.00 (0.00 to 2.81)
Renal insufficiency	9 (0.79%)	2 (1.3%)	0.63	1.64 (0.35 to 7.65)
Diabetes	72 (6.3%)	9 (5.8%)	1.00	0.91 (0.45 to 1.86)
Cerebrovascular disease (TIA or CVI)	35 (3.1%)	10 (6.5%)	0.06	2.17 (1.05 to 4.48)
ASA (calculated)			< 0.001	1.65 (1.30 to 2.09)
1	406 (36%)	37 (24%)		
2	537 (47%)	70 (45%)		
3	192 (17%)	48 (31%)		
4	1 (0.09%)	0 (0.00%)		
Type of Surgery			< 0.001	
Spine	206 (18%)	33 (21%)		1.22 (0.81 to 1.85)
Hip	413 (36%)	11 (7.1%)		0.13 (0.07 to 0.25)
Upper extremity	95 (8.4%)	3 (1.9%)		0.22 (0.07 to 0.69)
Knee	283 (25%)	101 (65%)		5.64 (3.95 to 8.06)
Foot	140 (12%)	7 (4.5%)		0.34 (0.15 to 0.73)

Footnote: Numbers are shown as mean (sd) or n (%)

^a^Alcohol intake, 1 “unit” corresponds to approximately 2 cl of liquor, 1 dl of wine or 3 dl of regular beer; BMI, body mass index

^b^Lung disease: multiple answers per patient were possible, e.g. presence of Asthma and a different severe lung disease in the same patient

*COPD* chronic obstructive pulmonary disease, *TIA* transient ischemic attack, *CVI* cerebrovascular insult, *ASA* American Society of Anesthesiologists

**Fig. 3:**
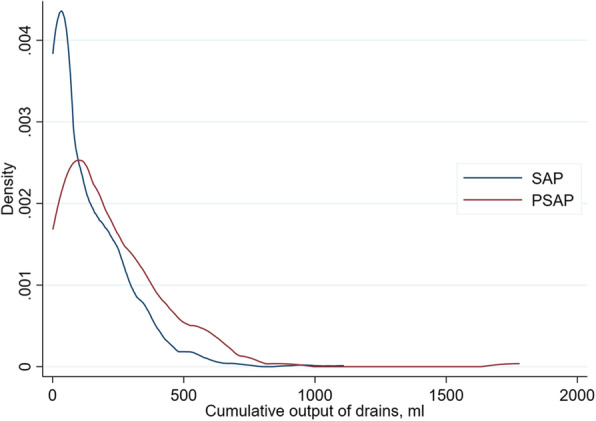
The cumulative drain output in millilitres in SAP vs PSAP patients is shown. If one patient had more than one drainage in surgical site, the total output of all drains was calculated. Note that the density function has no speaking unit, as it shows the proportion of values within a certain interval relative to all values

**Fig. 4:**
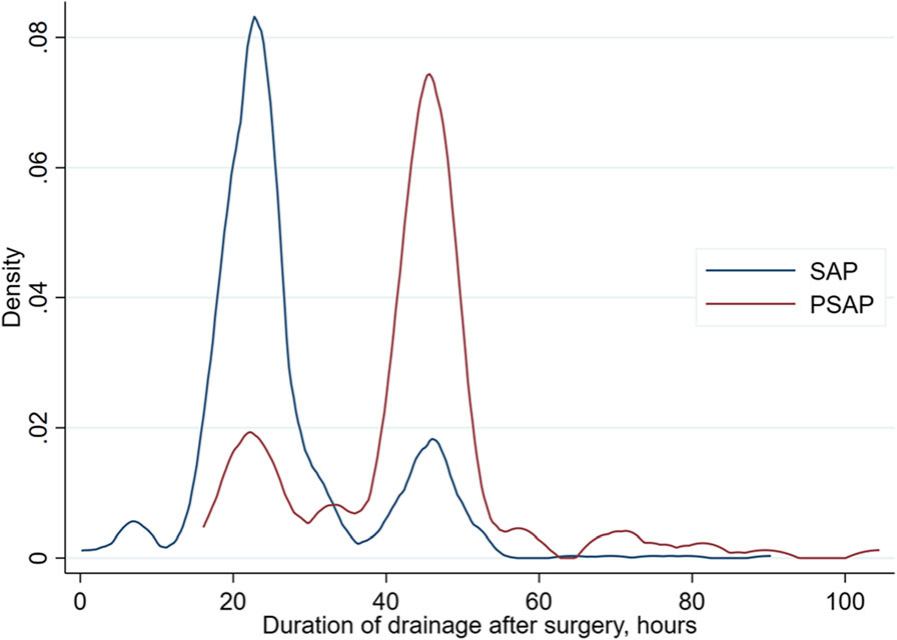
This figure shows duration of drainage after surgery in SAP vs PSAP patients. Note that the density function has no speaking unit, as it shows the proportion of values within a certain interval relative to all values

PSAP patients underwent prosthetic surgery (62% vs. 45%; *p* < 0.001) and knee surgery (65% vs. 25%; *p* < 0.001) more often compared to SAP patients (Tables 2 and 3). Indeed, prosthetic surgery was more strongly associated with PSAP with an OR of 3.57 (95% CI: 1.92 to 6.64), as was knee surgery with an OR of 5.64 (95% CI: 3.95 to 8.06). In prosthetic hip surgery 2.8% (10 of 361) were given PSAP whereas in prosthetic knee surgery PSAP proportion was 35.7% (85 of 238) (Table 4). 101 of 155 PSAP had knee surgery, with 16 not receiving any prosthetics. PSAP was also associated with a significantly longer duration of operation (87 vs. 68 min; *p* < 0.001; OR 1.01). The usage and number of drains at the surgical site (90% vs. 65%; *p* < 0.001 and 1.5 vs. 1.1; *p* < 0.001, respectively) as well as their cumulative output (224 vs. 131 ml; *p* < 0.001) were significantly higher in PSAP patients than in SAP (Table 1, Figs. 3 and 4). There was no difference in terms of post-operative transfusion of blood components.

**Table 3:** Surgical characteristics

Characteristics	SAP (***N*** = 1137)	PSAP (***N*** = 155)	***P***-Value	OR (95% CI)
Foreign material			< 0.001	
Prosthetic surgery	517 (45%)	96 (62%)		3.57 (1.92 to 6.64)
Metal or non-absorbable synthetic material	389 (34%)	47 (30%)		2.33 (1.21 to 4.48)
No foreign material	231 (20%)	12 (7.7%)		Reference
Surgeon ≥50 operations			< 0.001	
A	183 (16%)	4 (2.6%)		0.19 (0.05 to 0.68)
B	125 (11%)	2 (1.3%)		0.14 (0.03 to 0.70)
C	179 (16%)	7 (4.5%)		0.33 (0.11 to 1.03)
D	87 (7.7%)	51 (33%)		4.98 (2.00 to 12.4)
E	126 (11%)	19 (12%)		1.28 (0.48 to 3.39)
F	123 (11%)	7 (4.5%)		0.48 (0.15 to 1.51)
G	49 (4.3%)	16 (10%)		2.78 (1.00 to 7.67)
H	59 (5.2%)	0 (0.0%)		0.00 (0.00 to 0.45)
I	51 (4.5%)	6 (3.9%)		Reference
Duration of operation (minutes)	68 (41)	87 (45)	< 0.001	1.01 (1.01 to 1.01)
Type of anesthesia^a^				
ITN (intubation anesthesia)	449 (39%)	51 (33%)	0.13	0.75 (0.53 to 1.07)
SPA (spinal anesthesia)	557 (49%)	90 (58%)	0.040	1.44 (1.03 to 2.02)
LMA (larynx mask anesthesia)	76 (6.7%)	9 (5.8%)	0.86	0.86 (0.42 to 1.75)
Regional anesthesia	64 (5.6%)	9 (5.8%)	0.85	1.03 (0.50 to 2.12)
Others	20 (1.8%)	0 (0.00%)	0.16	0.00 (0.00 to 1.39)

Numbers are shown as mean (sd) or n (%)

^a^Type of anesthesia: multiple selection possible

**Table 4:** Type of surgery

Type of surgery	SAP (***N*** = 1137)	PSAP (***N*** = 155)	***p***-value	PSAP in %^**a**^
Spinal surgery (*N* = 239)	*N* = 206	*N* = 33	0.80	
Prosthetic surgery	0 (0.00%)	0 (0.00%)		
Metal or synthetics	172 (83%)	27 (82%)		13.6%
No foreign material used	34 (17%)	6 (18%)		15.0%
Hip surgery (*N* = 424)	*N* = 413	*N* = 11	1.00	
Prosthetic surgery	351 (85%)	10 (91%)		2.8%
Metal or synthetics	55 (13%)	1 (9.1%)		1.8%
No foreign material used	7 (1.7%)	0 (0.00%)		0%
Upper extremity surgery (*N* = 98)	*N* = 95	*N* = 3	0.52	
Prosthetic surgery	13 (14%)	1 (33%)		7.1%
Metal or synthetics	40 (42%)	1 (33%)		2.4%
No foreign material used	42 (44%)	1 (33%)		2.3%
Knee surgery (*N* = 384)	*N* = 283	*N* = 101	< 0.001	
Prosthetic surgery	153 (54%)	85 (84%)		35.7%
Metal or synthetics	32 (11%)	14 (14%)		30.4%
No foreign material used	98 (35%)	2 (2.0%)		2.0%
Foot surgery (*N* = 147)	*N* = 140	*N* = 7	0.70	
Prosthetic surgery	0 (0.00%)	0 (0.00%)		
Metal or synthetics	90 (64%)	4 (57%)		4.3%
No foreign material used	50 (36%)	3 (43%)		5.7%

^a^PSAP in %: percentage of PSAP patients per overall patients per parameter, e.g. prosthetic hip surgery 2.8% = 10 PSAP patients of overall 361 patients who underwent prosthetic surgery of the hip

Of all 30 orthopaedic surgeons involved, nine performed over 50 operations as the main surgeon and were evaluated concerning their frequency of PSAP use (Table 3). The proportion of PSAP use ranged from 0 to 33%. Crude odds ratios for PSAP ranged from 0.14 (95% CI 0.03 to 0.7; *p* = 0.017) to 5.0 (95% CI 2.0 to 12.4; *p* = 0.001) as compared to the reference surgeon. When adjusted for use of any foreign material (prosthesis, metal or non-absorbable synthetic material), the range of odds ratios was slightly larger and ranged from 0.13 (95% CI 0.03 to 0.66; *p* = 0.014) to 5.7 (95% CI 2.3 to 14.4; *p* < 0.001). The model when adjusted for foreign material fitted better than the crude model (*p* < 0.001).

SSI were infrequent (4 of 1292, 0.3%), only occurring in the SAP group (0 vs. 4; *p* = 1.000) (Table 1). All SSI were early infections within 30 days post-operative. Two patients with SSI underwent spine stabilization, one foot (removal of foreign material) and one knee surgery (primary TKA). Microbiological samples showed one infection with *Staphylococcus epidermidis* (spine), no germ (spine) and *Staphylococcus aureus* (foot). The patient with TKA matched criteria for a superficial SSI, without microbiological documentation.

## Discussion

SSI present a high burden for the patient which lead to increased mortality and morbidity, prolonged hospital stay, reoperations and higher costs [1–4]. Prevention of SSI is therefore essential. SAP is an effective measure to prevent SSI [8, 12, 20, 34]. Nevertheless, recommendations regarding the optimal duration of SAP remain controversial but several current guidelines are tending towards shorter periods [13–15, 35]. One must now address the problem of transferring the recently established recommendations into common practice [6, 36]. In this cross-sectional analysis we found the use of PSAP to be 12% at our centre and SSI proportions were 0.3% at 90 days. There were several patient- and surgery-related factors associated with it, with some of them being potentially modifiable. One strength of our study is that the heterogenous cohort represents a realistic patient selection found in daily surgical practice and includes a variety of orthopaedic operations, no exclusion criteria for comorbidities (apart from known infection before surgery) and no restriction for advanced age.

### Prolonged surgical antibiotic prophylaxis use

Our PSAP proportion of 12% was lower than reported in the literature. Although guidelines advise against PSAP, several studies have identified its use in daily practice. In one retrospective cohort study which included 34,133 patients undergoing major surgery, 59.3% of all patients and 63.7% (9280 of 14,575) of those undergoing THA/TKA were administered PSAP [6]. In THA/TKA the median duration of SAP was 39 h. In another retrospective observational study, which evaluated 1019 patients with TKA/THA, 21.7% of patients received PSAP [21]. One Swiss multicentre study of 2421 patients from all wards reported PSAP usage in 52.8% [22]. Two recent studies in Ethiopia also investigated SAP use and SSI rate. One retrospective study including 200 patients reported 90% post-operative SAP use and SSI proportions of 16% [37]. In contrast to our study, they included also contaminated wounds. The cohort was younger than ours (33 ± 15 vs 58 ± 14 years) and showed fewer known comorbidities (86% with none). The second study reported PSAP usage in 50.8% in patients undergoing different types of surgery, including orthopaedic, and an SSI proportion of 11.1% prior to discharge [38]. These two studies illustrate that a broad use of PSAP, even in young and healthy patients, does not facilitate SSI prevention.

### Predictors of prolonged surgical antibiotic prophylaxis

To our knowledge, few studies have investigated reasons for PSAP prescription. We found several factors associated with PSAP use, some of them being potentially modifiable (e.g. presence and number of wound drains, duration of surgery). In the literature, several of these factors are also reported to be risk factors of SSI: older age [23, 39], ASA score ≥ 3 [39–41], obesity [39, 41], longer duration of operation [39–41], presence of wound drain [41] and prosthetic surgery compared with other procedures (including non- orthopaedic surgery) [40]. Interestingly, a questionnaire for general surgeons on reasons for PSAP use revealed: drain use, fever, leucocytosis, avoiding conflicts with patients, and prescription for the surgeon’s own comfort and feeling of confidence [42].

In our cohort, a large proportion of SAP patients (65%, 740 of 1137) and an even higher percentage of PSAP patients (90%, 139 of 155) had at least one drain. In the literature, presence of drains and prolonged drainage were listed as potential risk factors for SSI [16, 43]. At our centre, drains were standardly removed on the first post-operative day after clinical visit by the operating surgeon. Individual decisions for alternative removal times were based on surgeons’ assessment. Several studies involving spinal surgery including drains found no difference in SSI proportions between SAP for the duration for which the drain was in site versus SAP for 24 h post-operative [29–31]. In addition, a meta-analysis and a more recent RCT found no benefit of drains in hip arthroplasty in connection with wound complications [44, 45]. In a related matter, our hip team completely abolished the routine use of drains during 2016. This could partly explain the difference in PSAP use in prosthetics of the hip (2.8%) versus knee (35.7%), but as the usage of drains was decreased gradually, no clear “before & after” analysis was possible.

As drains were associated with PSAP use in our study and do not seem to reduce surgical complications in the literature, we suggest minimizing their usage (e.g. drain reduction by means of hematoma reduction e.g. with use of tranexamic acid [46]).

In our study, indications for PSAP were not available, therefore, no conclusion about the importance or influence of one or several patient- or surgery- related factors on the decision-making process of the operating surgeon is possible. Nevertheless, our data indicates that surgeons have differing approaches, with some prescribing PSAP more often than others. Several studies investigating reasons for non-adherence to guidelines reported: surgeon mistrust in national guidelines due to perceived gaps in evidence, continuing habits and administered protocols to date. Other reasons included: institutional guidelines, fear of legal pursuit in case of complications under shortened SAP and communication problems between surgeons, anaesthetists and nurses involved in the SAP administration process [47, 48]. Therefore, preferences of the operating surgeon might influence duration of SAP.

The 0.3% SSI proportion at our centre was lower than reported in literature for orthopaedic surgery. Data from a 13-year multicentre SSI surveillance program in Swiss hospitals reported proportions of 1.6 and 1.3% in elective THA/TKA [5]. The following factors, also specified in the literature to reduce SSI, might have contributed to the low SSI proportions in our cohort: our voluntary participation in a Surveillance Program [5, 13, 49–51], having experienced and subspecialized orthopaedic surgeons, the inclusion of only elective procedures [52], as well as the use of SAP [8, 12, 20, 34]. This study does not allow a conclusion on the possible influence of post-operative SAP on SSI numbers.

Some SSI might have been missed due to the study design which assessed SSI via telephone interview at 30- and 90-days follow-up. A standardized clinical examination and a longer follow-up period (e.g. two years) to evaluate occurrence of late onset infection, could have enabled an optimal assessment.

Several current directives advise against any SAP in clean surgery without implantation of foreign material and a single pre-operative dose of SAP in other operations irrespective of prosthetic use [13–15, 35]. Addressing the shortening of SAP duration in orthopaedic surgery and remaining uncertainties regarding the safety for patients, Nagata et al. registered a multicentre, prospective trial comparing 24 h SAP versus prolonged SAP of 24 to 48 h in clean orthopaedic surgery (UMIN000030929) [53]. In addition, a RCT investigating peri-operative single- dose versus 24 h SAP in patients undergoing elective TKA was registered in 2017 (Clinicaltrials.gov: NCT03283878). Results of both studies are still pending.

### Limitations

A key limitation is the cross-sectional design of the study with retrospective survey of the use of SAP and bias inherent to this study design apply. Indications for PSAP use were therefore not available. However, the survey of PSAP use in a prospective way would have been comparable to an intervention and would have influenced results in our view. A further limitation is the lack of data on pre-operative SAP administration as they were not recorded in the electronic patient charts. Anyway, the focus of the study laid on investigating PSAP as pre-incisional SAP is well established.

## Conclusion

In conclusion, we found room for improvement concerning PSAP usage at our centre. Several potentially modifiable factors were associated with PSAP, including duration of operation or use of wound drains. Beside optimizing modifiable factors in order to reduce PSAP use, surgeons require high levels of evidence of optimized SAP resulting in low infection proportions with a high safety profile in order to alter their prescribing practices. In addition, a consistent continuation of programs for SSI surveillance is needed to homogenize common practice.

## Data Availability

The datasets used and/or analysed during the current study are available from the corresponding author on reasonable request.

## Abbreviations

SAP: Surgical antibiotic prophylaxis
SSI: Surgical site infection/−s
PSAP: Prolonged surgical antibiotic prophylaxis
CDC: Centers for Disease Control and Prevention
THA, TKA, TJA: Total hip, knee, joint arthroplasty/−ies
PJI: Periprosthetic joint infection
RCT: Randomized controlled trial
DECO- SSI: DECOlonisation and SSI (trial)
OR: Odds ratios
BMI: Body mass index
ASA classification: American Society of Anesthesiologists classification
NRS: Nutritional-Risk-Score
